# Unveiling the dynamic relationship of viruses and/or symbiotic bacteria with plant resilience in abiotic stress

**DOI:** 10.1007/s44154-023-00126-w

**Published:** 2024-02-05

**Authors:** Vasudha Sharma, Shakeel A. Mohammed, Nisha Devi, Gourav Vats, Hardeep S. Tuli, Adesh K. Saini, Yashika W. Dhir, Sunny Dhir, Bharat Singh

**Affiliations:** Department of Biosciences & Technology and Central Research Cell, MMEC, Maharishi Markandeshwar (Deemed to be University), Mullana, Ambala, Haryana 133207 India

**Keywords:** Infection, Microbes, Plants, Stress, Symbiosis, Virus

## Abstract

In the ecosphere, plants interact with environmental biotic and abiotic partners, where unbalanced interactions can induce unfavourable stress conditions. Abiotic factors (temperature, water, and salt) are primarily required for plants healthy survival, and any change in their availability is reflected as a stress signal. In certain cases, the presence of infectious pathogens such as viruses, bacteria, fungi, protozoa, nematodes, and insects can also create stress conditions in plants, leading to the emergence of disease or deficiency symptoms. While these symptoms are often typical of abiotic or biotic stress, however, there are instances where they can intensify under specific conditions. Here, we primarily summarize the viral interactions with plants during abiotic stress to understand how these associations are linked together during viral pathogenesis. Secondly, focus is given to the beneficial effects of root-associated symbiotic bacteria in fulfilling the basic needs of plants during normal as well as abiotic stress conditions. The modulations of plant functional proteins, and their occurrence/cross-talk, with pathogen (virus) and symbiont (bacteria) molecules are also discussed. Furthermore, we have highlighted the biochemical and systematic adaptations that develop in plants due to bacterial symbiosis to encounter stress hallmarks. Lastly, directions are provided towards exploring potential rhizospheric bacteria to maintain plant-microbes ecosystem and manage abiotic stress in plants to achieve better trait health in the horticulture crops.

## Introduction

The plant kingdom is one of the major divisions of an ecosystem where horticultural crops are extremely important to fulfill the continuous food demands of the ancient to the modern world. Changes in climate conditions and environmental factors including extreme temperature fluctuations, water availability, and variations in soil salt concentration are the components that have been considered as major abiotic stress-inducing factors. Usually, abiotic stress conditions even can be capable enough to affect the plant's health/immunity and jointly they prominently induce horrendous effects on plant growth (Gong et al. 2020). The impact of climate change on horticulture can also have ripple effects throughout food management, increasing food prices and reducing food availability for vulnerable populations (Srivastav et al. 2021). Subsequently, climate change can also exacerbate the spread of pests and diseases that can further damage crops and reduce yield. A more or less similar kind of effect must also be expected in the plants during the conditions of deprived or excess water availability. Whereas soil salt and nutrient imbalance could lead to altered plant growth, however, extreme physiological stress conditions can also cause unaccountable biochemical alteration in cellular pathways (Markham and Greenham 2021). Such signatures of abiotic stress appear as deformed and stunted growth patterns in the plants which need attention in terms of employing crop stress management policies to combat abiotic stress conditions in plants (Nepomoceno and Carniatto 2023).

**Fig. 1:**
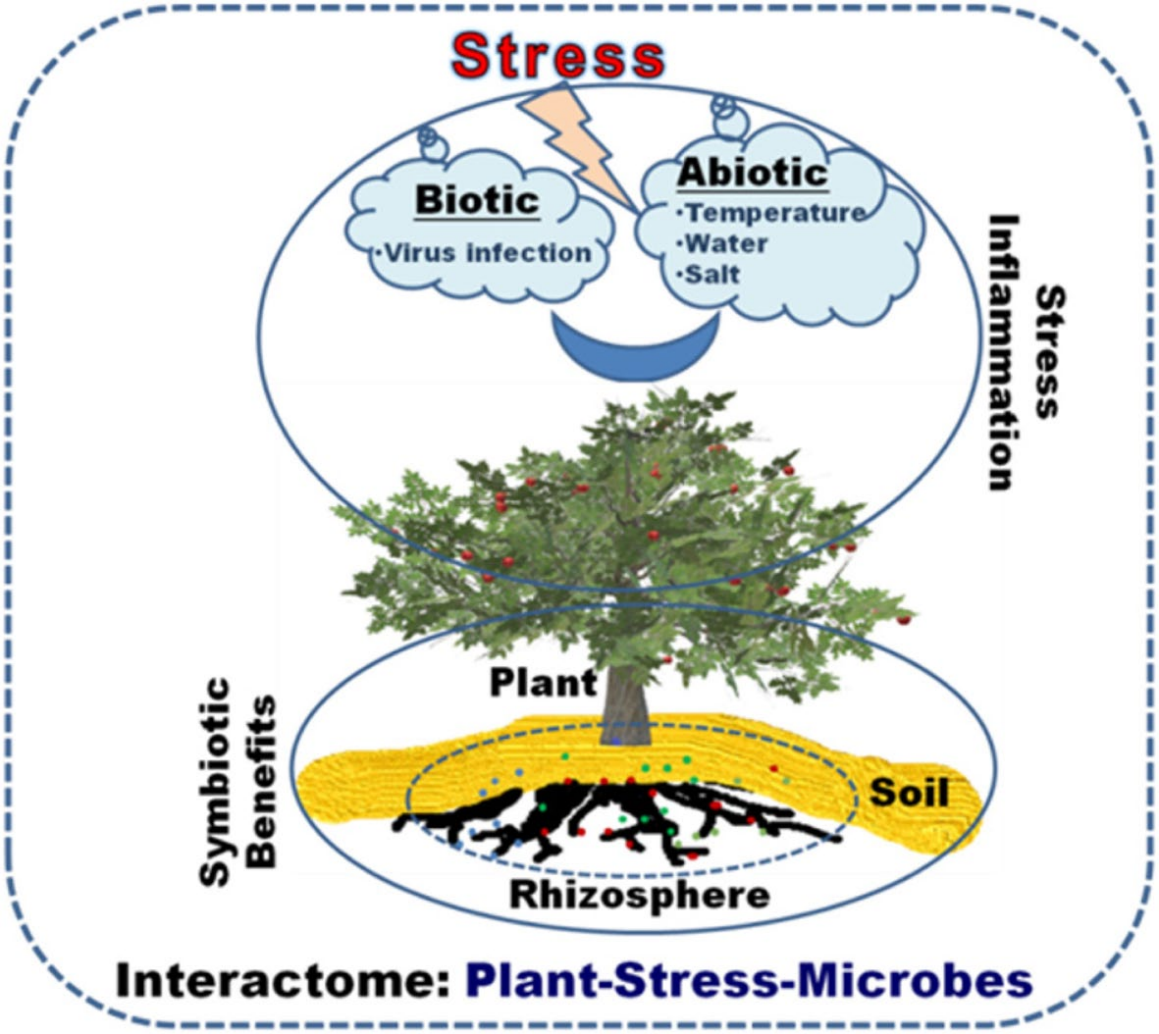
Illustration showing the interactions between plant microbe (viruses during infections and bacteria as root symbiont) during abiotic plant stress

On the other side, infectious plant viruses are also an important part of plants' ecosphere and hold a significant potential to alter crop productivity and yield. It has been noticed during abiotic stress conditions such as elevated temperature, the rate of virus replication and spread can lead to more severe infections and thus cause substantial crop damage (Rubio et al. 2020). Plant virus infections are a major concern in horticulture worldwide, causing significant losses in crop quality as well as yields (Falk and Nouri 2020). Some of the most common plant viruses include *Tobacco mosaic virus*, *Potato virus* Y, *Cucumber mosaic virus*, *Plum pox v*irus, *Cauliflower mosaic virus*, *Tomato yellow leaf curl virus*, and *Tomato spotted wilt virus* (Scholthof et al. 2011). These viruses are spread by insect vector or through mechanical means, and infect the seed material. The impact of plant virus infections on agriculture can be severe and could lead to reductions in crop productivity, loss of market value, and decreased food security. In some cases, plant virus infections can also result in the development of new virus strains/variants, further exacerbating the problem (Rubio et al. 2020). Plant viruses are often transmitted through insect vectors, and noticeably at the time of increased environmental temperatures, which also lead to increased insect populations, resulting in increased disease severity in plants. To combat plant virus infections at elevated temperatures, a variety of strategies can be employed, including the development of virus-resistant and heat-tolerant crops, the use of integrated pest management techniques, and the implementation of quarantine measures to prevent the spread of infected plants (Rubio et al. 2020). Additionally, international organizations and government agencies that are working to monitor should also respond to the plant viral outbreaks to minimize their impact on global food security (Calil and Fontes 2017). The damage caused by plant viruses in horticulture sector can be minimized through proper monitoring, prevention, and control measures. Furthermore, attention to both abiotic and biotic (virus infections) stress must be required simultaneously for better stress management in plants (Ray and Casteel 2022). When plants are exposed to both the stresses together, they interact in various ways. Plants continuously secrete several metabolites that serve a variety of direct and/or indirect benefits; including innate immunity, defence response signaling, plant growth and development, response to environmental stresses, warding off pests and pathogens, promoting plant-microbe symbiosis, and modifying microbial communities associated with hosts biosphere (Erb and Kliebenstein 2020). There are several reports where virus-infected plants become more resilient to abiotic stresses like drought, salinity, etc. In addition, plant microbiomes are also crucial for the development of immunity, disease suppression, nutrition supply, and defence against abiotic and biotic stresses in plants (Haldar and Sengupta 2015). A diversity of secondary metabolites (SMs) has been produced by plants that grow in different areas and/or fluctuating growth conditions (Mishra et al. 2021). An interesting question is whether the production of such SMs could be able to dictate the diversity of plant rhizosphere-associated bacterial symbiotic consortia. If yes, then bacterial association and cross-talks between the plant and bacterial molecules have the potential to encounter abiotic stress outcomes in the plants and could be able to improve crop health in all respects (immunity, yield, and nutrition values). Conceptually, abiotic stress, virus pathogenesis (biotic stress), and microbial symbiosis are not easy to relate together due to the coherence and interference of innumerable factors that are undergoing continuously in plants during such multipartite associations and stress conditions. Therefore, for a comprehensive understanding, it is essential to delve into the intricate cellular and molecular interactions that take place between plants, pathogens, and symbionts. Such information could be useful to understand multipartite associations of plants during normal as well as stress conditions. Therefore, with the excitement to know more about the curious and furious fact of abiotic stress with overlaps of infectious virus-induced biotic stress, their respective and/or cumulative effects on the plants are discussed as the first major section of the article. Whereas normal and concurrent interactions during abiotic stress of plant with root symbiotic commensal bacteria are thoroughly discussed in the second section of the article in light of recent and relevant literature. An overview of viral pathogen-plants-symbiotic root microbes has also been shown in Fig. 1.

## Abiotic stress and virus infections in plants

Abiotic stress exerts an unconstructive impact of lifeless factors on livelihood. These include temperature, drought, salinity, and other extremities that play a pivotal role in horticulture as they affect various stages of crop growth and development (He et al. 2018). Ideal temperatures for plants vary depending on the species, but in general, warm temperatures promote germination, growth, and flowering, while extreme temperatures can lead to stunted growth, reduced yields, and even death (Bita and Gerats 2013). Additionally, temperature affects soil and water conditions, which in turn affects plant growth and productivity (Seleiman et al. 2021). Hyper-salinity and drought are among the primary causes of crop loss worldwide. In general, a suitable range of temperature, salt, and water is required to sustain optimal growth and yield of crops (Ma et al. 2020). All these abiotic stresses influence plant growth in natural and diseased conditions. However, certain common host factors play an important role during stress induced by high temperatures, salinity, and drought with biotic stress like viral infections (Audil et al. 2019). 

Viruses are intracellular, obligate pathogens that affect almost every biological entity present on the planet (Guo et al. 2019). Plant virus infections are a major concern in agriculture worldwide, causing remarkable losses in the quality and yields of crops. Some of the most common plant virus infections include that caused by *Potato virus X*, *Tobacco mosaic virus*, *Potato virus Y*, *Cucumber mosaic virus*, *Tomato yellow leaf curl virus*, *Cauliflower mosaic virus*, *Plum pox virus*, *Brome mosaic virus*, and *Tomato spotted wilt virus* (Scholthof et al. 2011).

Abiotic factors like temperature, salinity, and drought play pivotal roles in shaping the virus's evolution, virus spread, eradication, etc. (van Munster 2020). On the contrary, plant viruses can have a significant impact on crop productivity and yields, particularly during different abiotic stresses. Warmer temperatures can increase the rate of virus replication and spread, leading to more severe infections and greater crop damage (Velásquez et al. 2018). However, in some cases, higher temperatures sustain the viral infection but over time lead to decreased accumulation of viral load (Roberts et al. 2018). Drought usually stimulates plant virus infections, further exacerbating the problem (Seleiman et al. 2021). Salinity stress was also demonstrated to favour the propagation of viral pathogens within the plants (Prasad et al. 2022). In the subsequent sections, we have discussed the role of various abiotic stresses on plant viral diseases. An overview of the defensive systems, and regulatory network affected by multi-stress (virus infections and abiotic) conditions is presented here with practical potential that could deliver resilience, resistance, and subsequently higher yield.

Many abiotic stresses affect the overall plant growth like temperature, drought, heavy metals, ultraviolet radiations, and salinity (He et al. 2018). Among these, three stress factors i.e., temperature, drought, and salinity hugely affect plants that either prone them to viral infections or make plants resilient towards them. A few such important stress factors affecting plant mechanisms are listed in Table 1.

**Table 1:** Interactions of plant viruses with plant during the conditions of abiotic stress

**Abiotic stress**	**Virus name**	**Host plant**	**Target mechanism**	**References**
**1. High Temperature**				
** (i)**	*Tobacco mosaic virus (TMV)*	*N. tabacum*	• Higher temperature (28 °C), suppresses hypersensitivity response via N gene targeting • Inhibit NADPH oxidase and other oxidase genes	(Király et al. 2008)
** (ii)**	*Tomato spotted wilt virus* (TSWV)	*Capsicum* *chinense,* *Datura stramonium,* *N. tabacum,* *Physalisixo carp*	• At 32°C break down of plant resistance via targeting of heterozygous *Tsw* gene locus that ultimately causes death • *Physalisixoc arp* and *Datura stramonium* show faster symptom development at 29 °C	(Moury et al. 1998)
** (iii)**	*Tomato ringspot virus* (ToRSV)	*N. benthamiana*	• vsiRNAs infected plants show signs of illness comparatively at 27°C than 21°C. After 8 days of infection at 27°C sickness disappear and movement protein and coat protein were present at very low levels in young leaves	(Ghoshal and Sanfaçon 2014)
** (iv)**	*Barley yellow dwarf* *virus -*PAV (BYDV-PAV)	*Triticum aestivum* *cv.* Yitpi	• At 21°C virus replicate rapidly multiply easily which led to appearance of early signs of virus with high viral titre	(Nancarrow et al. 2014)
** (v)**	*Groundnut bud necrosis virus* (GBNV)	*Vigna unguiculate*	• At 25°C H_2_O_2_ build up quickly and cause faster leaf death	(Singh et al. 2018)
** (vi)**	*Turnip mosaic virus* (TuMV)	*Brassica campestris*	• Viral coat protein synthesis and accumulation are more rapid at high temperature Rate of systemic infections up to 23°C increases as the temperature is increases.	(Chung et al. 2015)
** (vii)**	*Tomato yellow leaf curl virus* (TYLCV)	*S. lycopersicum*	• A TYLCV infection worsen the heat stress dealing in plants • Viral protein stops HSFA2 movement to nucleus that shuts down HSF-regulated genes (Hsp17, Apx1, Apx2, and Hsp90) leading to slow stress reactions • By infecting a plant with TYLCV response to heat stress regulated by HSP90 and SGT1 gets turned off	(Anfoka et al. 2016) (Moshe et al. 2016) (Gorovits et al. 2019)
** (viii)**	*Potato virus Y* (PVY)	*S. lycopersicum*	• At 28°C plants become more sensitive because of decreased activity of enzymes involved in the methionine cycle (methionine synthase (MS, SAH, SAM)	(Fesenko et al. 2021)
** (ix)**	*Cotton leaf crumple virus* (CLCrV)	*Gossypium hirsutum*	• Better virus silencing at high temperatures (30 °C /26 °C) than low temperatures (22 °C /18 °C). • At 30 °C and 26 °C automatous gene silencing is less effective.	(Tuttle et al. 2008)
** (x)**	*Turnip crinkle virus* (TCV)	*A. thaliana*	• Infected plants protect from viruses by altering the RNA silencing pathway via turning up the genes (DCL2, AGO2, HEN1, RNA methyltransferase)	(Zhang et al. 2012)
** (xi)**	*Lettuce chlorosis virus* (LCV)	*N. benthamiana*	• At high temperature viral suppressor protein P23 enter into the leaves via agro-infiltration and causes severe local necrosis.	(Kubota and Ng 2016)
** (xii)**	*Peanut stunt virus* (PSV)	*N. benthamiana*	• As viral RNA and satellite RNA quickly build up in the early stages of an infection, a high temperature (27°C) is linked to the early onset of disease symptoms. • Proteins (chaperonin 20, peptidyl-prolylcis-trans isomerase, and peroxiredoxin) are predominant in infected plants as compared to uninfected one	(Obrępalska-Stęplowska et al. 2015)
**2. Salt**				
** (i)**	*Cowpea severe mosaic virus* (CPSMV)	*Vigna unguiculata*	• Salt stress favor viral particles to multiply that resulted in reduced plant size, photosynthetic rate, chlorophyll, and carotenoids	(Varela et al. 2019)
** (ii)**	*Potato virus A* (PVA)	*N. benthamiana*	• Abiotic stressors increased the expression of viral genes by increasing cytosolic calcium • Spreading of PVA is quick due to calcium flux caused by salt stress in cytosol	(Suntio and Mäkinen 2012)
** (iii)**	*Flock house virus* (FHV) [insect virus]	*N. tabacum*	• Transgenic plants with more FHVB2 (RNA silencing suppressor) have more chlorophyll and can grow seeds better at 42°C By storing more proline such transgenic plants are capable to tackle high salt concentration	(Sinha et al. 2021)
**3. Drought**				
** (i)**	Cucumber mosaic virus (CMV)	*Arabidopsis thaliana*	• The CMV encoded -RNA silencing suppressor protein 2b makes 2b – transgenic pants more resistant to drought by inhibiting ABA regulated gene induction	(Westwood et al. 2013)
** (ii)**	*Turnip mosaic virus* (TuMV)	*Arabidopsis thaliana*	Drought-adapted viruses make plants more resistant to drought by altered expression of host genes. Some of them are involved in circadian clock, hormone production and growth signaling pathways.	(González et al. 2021)
** (iii)**	*Tomato yellow leaf curl virus* (TYLCV)	*Solanum lycopersicum* *N. benthamiana*	• Without ABA, C4 viral protein present in plasma membrane makes plant more resistant to drought. • Plants with viral infections showed delayed signs of wilting and mild signs of drought. TYLCV infection made drought-induced osmoprotectants more stable (like sucrose and glucose; amino acids such as proline, valine, and tryptophan and HSPs) • TYLCV-infected plants better survive in harsh drought conditions. • TYLCV infected plants survived in drought by showing signs of the infection later than usual.	(Corrales-Gutierrez et al. 2020; Shteinberg et al. 2021)
** (iv)**	*Brome mosaic virus (BMV), Cucumber mosaic virus (CMV), Tobacco mosaic virus (TMV), Tobacco rattle virus (TRV*)	*Oryza sativa, N. benthamiana, Beta vulgaris, Capsicum annum*, *Cucumislanatus, Cucumissativus, S. lycopersicum, Solanum habrochaites, Cucurbita pepo*	• When plants were infected with CMV or BMV, they made more osmoprotectants (trehalose, putrescine, and proline) they also synthesize more salicylic acid, antioxidants and sugars that are linked with augmented drought tolerance	(Xu et al. 2008)
** (v)**	*Cauliflower mosaic virus* (CaMV)	*A. thaliana*	• Viral infection is controlled by the stress of water scarcity. • In water deficiency, virus-transmission rate enhanced in some case but decreased in others.	(Bergès et al. 2018)

### Plant-virus interactions during temperature stress

Plants compete strongly in an environment those have a sympathetic environmental temperature. Plant growth and development are unnatural with fluctuations in optimum temperature that directly affect their cellular morphology and cellular signalling (Hatfield and Prueger 2015). Elevated temperature impacts the plant morphology and shows phenotypic effects from sweltering of stem and leaves, shortening of life process, ablation of plant parts, inhibited growth of roots and shoots, sexual cells becoming sterile, and also affecting fruit quality. During higher temperatures, the physiology of cells also gets affected and is mainly characterized by a reduction in photosynthetic and respiratory rate, membrane permeability and fluidity changes, etc. (Desaint et al. 2021).

Temperature plays an important role in overall plant development and higher temperatures are known to act as pro-viral factors in the early stages of infection with virus eradication in later stages. Infection of *Capsicum chlorosis virus* promoted viral replication at the commencement of infection and recovered systemically at the subsequent stages because of vigorous incitement of RNA silencing machinery in the host (Tsai et al. 2022). High viral accumulation of Peanut stunt virus was recorded at higher temperatures (27 °C) that decreased tremendously later, contrary to high viral accumulation both at initial and later stages of infection at 21°C (Obrępalska-Stęplowska et al. 2015). Replication of *Barley yellow dwarf virus (BYDV)* was boosted at a higher temperature (21 °C), which also caused the emergence of early virus symptoms and a high viral titer in *Triticum aestivum cv*. *Yitpi* (Nancarrow et al. 2014). In general, there are two possible outcomes in different host-pathogen combinations about heat stress, 1) either the virus accumulates largely, or 2) the host recovers from infection. Some common regulatory mechanisms were highlighted when plants were subjected to both heat and viral infections. Proteome analysis highlighted a significant decrease in the accumulation of proteins participating in photosynthesis (like rubisco, ferredoxin-NADP reductase) and carbohydrate metabolism (like Fructose-1,6bisphosphatase) at different temperatures (Obrępalska-Stęplowska et al. 2015). However, heat-stressed plant species have similar proteins affected (Zhao et al. 2018). It has been reported that there are elevated levels of heat shock protein (HSPs) dynamics in potato plants when they were subjugated to either warmth or to *Potato virus Y* (PVY) infection (Table 1). Investigations yet again decorated that PVY infection and torrid heat stress have assured the existence of common regulatory mechanisms (Makarova et al. 2018). These findings have important implications because plants respond by changing protein expressions to stresses such as heat and viral infections which is a plant's adaptation behaviour. Subsequently, the accumulation of these proteins affects the virus titer and hence infection.

Some reports have mentioned that host resistance is altered under increased temperature (Desaint et al. 2021). Resistance is heritable immunity that minimizes damage induced by plant pathogens. During the interaction of *Nicotiana xanthi* and *Tobacco mosaic virus* (TMV), the hypersensitive response (HR)-type resistance was overcome when plants were kept at higher temperatures. This was evidenced by the downregulation of superoxide and other antioxidant enzyme activity that were associated with the suppression of HR-type resistance (Király et al. 2008). It was also reported that infection of TMV in *N. tabacum* increased with increasing temperature to 28◦C. In another report, *Tomato spotted wilt virus* (TSWV) and *Potato virus X* (PVX) co-infection, the procedure of HR abolishment was similar and quite noticeable (Wang et al. 2009; Chung et al. 2018). It was implicated that temperature fluctuations either change R-gene protein conformation or the intrusive virus destabilized R-genes at higher temperatures promoting virus multiplication. It has been reported that elevated temperatures lead to the denaturation of existing proteins or the misfolding of newly synthesized proteins (Volkening et al. 2019). *Solanum lycopersicum* HSPs interact with TYLCV proteins and promote virus accumulation. HSPs are expressed in response to heat so that mature proteins are folded appropriately to perform necessary functions. The segregation of HSPs between virus and heat strain retaliation led to incompetent implementation of the protein due to that heat stress response efficiency was decreased as detected in *TYLCV*-infected tomatoes prone to high temperature (Prasad et al. 2019a). High temperatures and dryness dramatically boosted Arabidopsis' sensitivity to TuMV by inhibiting PR and R genes (Prasch and Sonnewald 2013). It has been demonstrated that the NBLRR (N) protein, which detects the TMV signal, underwent a conformational shift after TMV infection. As a result, plant cell death (PCD) that limits viral replication and propagation was not able to start the signal transduction chain. The infection altered the N gene's role in resistance because less N protein accumulated in the nucleus, which inhibited downstream signalling connected to hypersensitive response suppression. In the course of TMV infection of tobacco and *Tomato Spotted Wilt Virus* (TSWV) infection of tomato, HR response and R-gene mediated plant defence responses dependent on heat has become reduced (Zhu et al. 2010). High temperature (32°C) has also been shown to disrupt capsicum and tabacum plant resistance to TSWV mediated by heterozygous Tsw gene locus, causing systemic dissemination and the emergence of necrotic signs. Hence, in some virus-host combinations, changes in hosts are induced by viruses for their survival (Table 1) (Llamas-Llamas et al. 1998; Moury et al. 1998).

Viruses are obligate parasites and are dependent on host factors for their survival. Their infection leads to the activation of a defence pathway, known as RNA silencing, at the viral entry (Prasad et al. 2019a). The double-stranded RNA replication intermediates are targeted by a class of endonucleases known as DICERs that target them and reduce them to 21-24nt sized small interfering RNAs. These siRNAs bind to their cognate viral RNAs and silence them. RNA silencing response is suppressed under high temperatures and increases plant susceptibility to pathogen infections (Travella et al. 2006). Many studies indicated depletion in viral titer in the new leaves due to the accumulation of siRNAs (Sahu et al. 2010).

The generation of these virus-induced siRNAs is affected by temperatures that consequently enhance/reduce the viral load*. N. benthamiana* plants infected by *Tomato ringspot virus* (ToRSV) displayed symptoms earlier when kept at 27 °C compared to 21°C, due to the effective generation of vsiRNAs. However, plants recovered from infection 8 days post inoculation (dpi) at 27°C, with symptomless leaves containing fewer viral proteins (Ghoshal and Sanfaçon 2014). In viral infections of the fruit trees, it has been shown that low temperatures favour the viral infection as the host's silencing mechanism is arrested at lower temperatures (Tatineni et al. 2009). *Brassica campestris* plants infected with TuMV when kept at a high temperature accumulate more viral coat protein. Up to 23°C, rising temperature causes a proportional increase in the rate of systemic infection (Chung et al. 2015). Contrarily, *Cotton leaf crumple virus* (CLCrV) infection in *Gossypium hirsutum* plants displayed enhanced virus silencing when plants were kept at higher temperatures (30°C/26°C day/night), even though endogenous silencing of the gene is diminished at 30 °C and 26°C (Table1) (Tuttle et al. 2008).

With the recent advancements in biotechnology, plant virus infections are now reported to endow plants with properties that allow them to combat different stresses, though in general, it has been discovered that pathogenic infections reduce a plant's ability to withstand stress due to abiotic factors (Márquez et al. 2007). For example, tomato plants with ToYLCV infection were sensitive to high temperatures to a greater extent. Reduced responsiveness of HSFs and HSPs has been linked to this enhanced heat vulnerability (Anfoka et al. 2016). In another report for illustration of this mutualistic interaction, the *Pigeon pea sterility mosaic virus* (PPSMV)-I and -II-infected pigeon pea plants have high-temperature stress tolerance (Kumar et al. 2017). It was claimed that *B. vulgaris* with CuMV infection had improved cold tolerance (Xu et al. 2008). Earlier it was believed that viruses were just parasites that harmed plants and reproduced by utilizing the resources and chemicals of their hosts. The positive functions of some viruses for their plant hosts, however, have been detailed by fresh information in recent years. According to one definition, viruses interact mutually with plant cellular systems, providing some abiotic stress protection for plants (González et al. 2020). One of the early research in this field demonstrated how RNA viruses like the *Cucumber mosaic virus* (CMV) could make plants resistant to cold and drought (Xu et al. 2008). Abiotic stress is also exerted at high temperatures which can further result in an elevation in the occurrence of insect vectors that carry with them a load of pathogens. With the increasing global temperatures, increasing the yield by the development of plants that can withstand infections and higher temperatures is necessary to meet increasing food demand.

### Plant virus interaction during drought stress

Drought is a serious abiotic stress that affects plant growth and proportionally crop output (Franklin et al. 2016). It is one of the primary plant stressors, affecting fitness or sometimes even leading to mortality depending on its intensity and duration (Xu et al. 2008). The stress induced by water not only interferes with plant-virus interactions but also affects plant-insect interactions, which further exacerbates virus spread (Davis et al. 2015; Blanc and Michalakis 2016). Drought and pathogen stress have been found to be detrimental to plant growth and productivity (Sinha et al. 2016). Pathogen infection has also been demonstrated to influence plant responses to water scarcity. Drought can have a good impact and reduce illness levels, but it also increases disease susceptibility in many circumstances (Prasch and Sonnewald 2013; Ramegowda et al. 2013; Kissoudis et al. 2014). The virus-infected plants may have reduced basal defences that could make them more susceptible to drought stress (Hulten et al. 2006). For instance, when Sweet corn plants infected with *Maize dwarf mosaic virus* (MDMV) were concurrently exposed to drought stress, they demonstrated a greater drop in leaf area, ear weight, and height of plant than non-infected plants (Wang et al. 2012).

Several studies also reported beneficial effects of virus infection that make plants resilient to drought stress. In one such case, Arabidopsis plants infected with TuMV, were passed through serial passages that induced virus evolution, under normal watering conditions and separately under drought stress. The results demonstrated that the drought-evolved TuMV increased Arabidopsis efficiency to withstand drought tolerance. The findings demonstrated how a virus adapted to stress conditions and subsequently benefitted the host plant. Recent findings also highlighted the induction of more drought tolerance in plants with serious virulent infections and modest tolerance with superficial viral infection, allowing the plant to complete its life cycle and produce viable offspring. In addition, virus infections minimize water loss through reduced transpiration due to partial stomatal closures (Xu et al. 2010). It was anticipated that higher viral-induced tolerance is invariably followed by improved biological fitness (Aguilar et al. 2017). Interestingly, TYLCV infection in tomato plants where virus-infected plants were more resilient when compared to uninfected plants (Mishra et al. 2022). Virus-infected plants under drought stress have boosted resistance mechanisms compared to non-infected plants. Co-infection of *Potato virus X* (PVX) and *Plum pox virus* (PPV) in *N. benthamiana* and Arabidopsis displayed improved drought tolerance by boosting salicylic acid levels further improving photosynthetic performance and enhanced activity of antioxidant enzymes (Khalvandi et al. 2021). *N. benthamiana* plants infected with *Brome mosaic virus* (BMV), *Cucumber mosaic virus* (CMV), and TuMV revealed belated emergence of leaf drooping and stem dryness compared to non-infected drought-stressed plants (Gentleman et al. 2004). The infected plants accumulated more osmoprotectants such as glucose, fructose, and sucrose (Xu et al. 2010). The enhanced amounts of these metabolites in virus-infected plants primed plants to cope with abiotic stress. The metabolic and physiological changes caused by viral infection may have mitigated the impacts of drought stress and hence conferred combined stress tolerance.

### Plant virus interaction during salt stress

Abiotic stressors have a significant impact on plant growth and yield (Gharsallah et al. 2016). High salt concentrations in saline soil prevent plants from absorbing water and nutrients (Gong 2021). Due to this imbalanced osmotic stress and ionic stress are induced (Zhu 2002). Collectively, physiological and molecular changes brought on by salt stress prevent plants from growing by lowering the amount of resources available, limiting photosynthesis, and suppressing cell division and growth (van Zelm et al. 2020). Excess sodium-induced changes in calcium levels and reactive oxygen species (ROS) are early indicators that trigger a salt stress response (Park et al. 2016). Plant encountering salt stress enhances the cytosolic calcium level due to ion and osmotic stress brought on by salt stress. Calcium activates Ca^2+^ sensors and further serves as a crucial secondary messenger (Yuan et al. 2014; Zhang et al. 2020).

Plant viral infections have different outcomes in virus-host interactions under salt stress. According to some recent findings, the host plant's viral load rises as a result of the plant's cells sensing and response to abiotic stress. As a result, increased field crop losses could result from the interaction of abiotic stress and viral infection (Zhao et al. 2021). In viral infections, calcium has been shown to play a role in virion generation, maturity, and stability, as well as viral entrance and replication. Whether through pumps and/or channels on the plasma membrane, viral proteins regulate calcium flux and also cause calcium release from internal reserves (Zhou et al. 2009). Several viral proteins trigger calcium-responsive transcription by interacting with cellular calcium receptors. In potatoes infected with *Potato virus Y* (PVY), the transcript levels of genes related to photosynthesis, perception, signalling, and defensive responses were changed (Baebler et al. 2009). At the onset of infection in susceptible potatoes, genes involved in calcium-signalling were up-regulated, suggesting that calcium may be crucial for the development of potyvirus infection. In another report on *Pepper yellow mosaic virus* (PepYMV) infections, it was found that 4.9% of the genes up-regulated were related to calcium-mediated signal transduction (Alfenas-Zerbini et al. 2009). Salinity stress has been shown to encourage the expansion of bacterial and viral diseases inside plants, and increased rate of infection (Kissoudis et al. 2016; Varela et al. 2019).

Recent findings reported the resistant-breaking mechanisms in plants under salt stress. In tomato TYLCV combination, regardless of whether the tomato genotypes were virus susceptible or not, salinity stress caused a considerable viral accumulation. The sensitivity of the tomato-susceptible genotype to TYLCSV was significantly increased, and its defence appeared to be weaker. The efficacy of the Ty-1, Ty-3, and Ty-5 loci-mediated TYLCSV resistance was significantly reduced by salt stress. Factors related to salinity decreased the efficiency of the tomato TYLCSV resistance. An impervious genotype of cowpea when treated with salt stress and then infected with *Cowpea severe mosaic virus* (CpSMV) has exhibited critical symptoms. It was hypothesized that salinity-related disturbances in the redox metabolism contributed to the virus's more severe symptoms (Varela et al. 2019). Furthermore, it was discovered that *N. benthamiana* plants were more vulnerable to *Potato virus A (PVA)* infection when exposed to salinity stress (Suntio and Mäkinen 2012). It was also been reported that during the conditions of salt stress, seedlings of *N. benthamiana* obtained from mock-inoculated plants depict elevated inhibition of root growth as compared to those that were infected with PVX *(Potato Virus X*). Furthermore, corresponding fresh weights of progenies derived from PVX were enhanced by 1.6 fold when compared with those which were mock inoculated in the presence of NaCl and Mannitol respectively (Hernández-Walias et al. 2022).

One of the main approaches utilized by viruses to create favourable interactions with plant hosts is a reduced rate of photosynthesis, which further reduces the accumulation of ATP and NADPH required for plant defence (Bolton 2009). Such defence-compromising mechanisms induced by salt stress, result in the loss of viral resistance in plants. More severe symptoms of the virus have been proposed to be caused by salinity-related changes in redox metabolism (de la Torre et al. 2014).

**Fig. 2:**
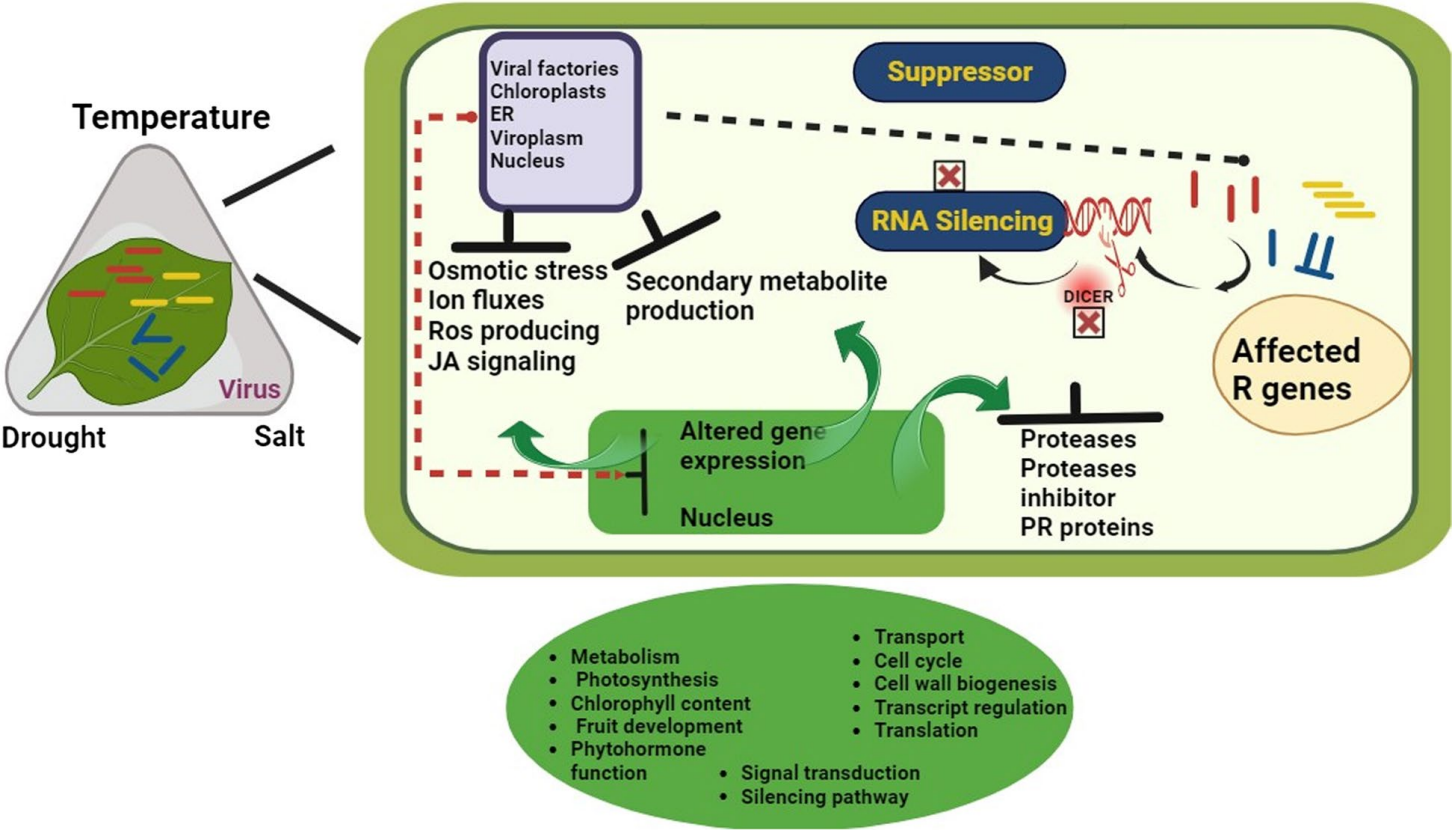
Effect of multipartite associations (virus-abiotic stress) on plants and affected cellular processes

Contrary to the increased susceptibility of plants to viral infections under salt stress, resistance/tolerance has also been imparted in a few cases. Systemic *Eggplant mottled crinkle virus* (ECMV) accumulation was dramatically reduced in *N. benthamiana* plants when they were exposed to salt stress before infection. In another such example, the overexpression of an insect Flock house virus B2 protein increased the ability of rice and tobacco to resist salt stress by enhancing stomatal performance and photosynthesis (Table 1) (Sinha et al. 2021). The connection between biotic and abiotic stress factors and plant responses possibly hypothesized the existence of common defence routes for plant adaptation to challenging situations (Moldakimova et al. 2012) (Fig.2).

## Plant-symbiotic bacteria interactions during abiotic stress

### Plant-symbiotic bacteria interactions during temperature stresses

High temperatures are a type of usual stress in plants where conditionally several root associate soil microbes conduct a molecular cross-talk to provide dictations for survival adaptations. Every plant-pathogen interaction has a disease-causing temperature range such as *Globodera pallida* nematodes infect the potato plants at 15 °C (Jones et al. 2017) whereas *Xanthomona soryzae* colonize in the plants at 35 °C as well as 27 °C (HORINO et al. 1982). However, such kind of temperature variations and the infectivity potential of bacteria (beneficial and/or harmful) are difficult to predict. It has been noticed that effectors triggered immunity (ETI) activation at higher temperatures reduce plant defence hormone salicylic acid (SA) and SA-associated defence gene expression, making Arabidopsis more susceptible to pathogens such as *Pseudomonas syringaepv* than Tomato (Li et al. 2020). Temporary heat stress decreases the pattern-triggered immunity (PTI) signalling and Pst DC3000 resistance in Arabidopsis (Cheng et al. 2013; Janda et al. 2019). PTI-responsive genes, MAPK, and BIK1 phosphorylation were also be reported to activated more strongly even after a brief exposure to increased temperature (Cheng et al. 2013). Heat stress can influence unstressed progeny's immunity through epigenetics (Liu et al. 2019). High temperatures suppress type IV secretion-associated pilus formation and virulence gene expression in *Agrobacterium* infections (Jin et al. 1993; Baron et al. 2001). At higher temperatures, *Pectobacterium atrosepticum* produced more plant cell wall-degrading enzymes, quorum-sensing signals, and disease development (Hasegawa et al. 2005). Microorganisms with altered respiration grow faster at high temperatures which could increase microbial soil respiration (Karhu et al. 2014). Soil microbes use organic matter in a temperature-dependent manner (Frey et al. 2013). Soil warming and drought indirectly affect rhizosphere nutrient uptake and carbohydrate exchange (Newsham et al. 1995). Rhizosphere bacteria and endophytes reduce plant temperature stress and promote crop growth in different climates, soils, and temperatures. However, soil and temperature status may affect plant-beneficial bacteria interactions (Bilal et al. 1993; Javed and Arshad 1997; Bashan 1998). *Mycobacterium sp. 44*, *P. fluorescens*, and *Pantoea agglomerans* strains isolated from a semi-continental climate significantly increased winter wheat root and shoot growth at 16°C compared to 26°C. *M. phlei strain MbP18* and *Mycoplana bullata MpB46*, both from semi-arid climates, performed well under both conditions, indicating genotype-specific environmental preferences (either loamy or sand). It was demonstrated that desert woody legume *Prosopisgla ndulosa* nodule *Rhizobia* grows better at 36°C than the 26°C (Waldon et al. 1989). Bacteria colonizing different sites may also react differently to environmental conditions. *Burkholderia phytofirmans PsJN* showed a temperature increase from 10 to 30°C. Reduced tomato rhizosphere colonization of this strain, but endophytic abundance remained unaffected (Pillay and Nowak 1997). Rhizosphere and endophytic bacteria can induce a systemic response to reduce plant stress from temperature or drought (Lichtfouse 2009; Yang et al. 2009). Heterotrophs' carbon incorporation has been noticed to be increased even at a little rise (40 °C) for a short time, which possibly could be due to phytoplankton's instant release of photosynthate. Attachment and higher temperatures accelerate heterotrophic incorporation of phytoplankton-derived dissolved organic carbon (DOC). Warmer temperatures double the DOC incorporation by free-living heterotrophs and quadrupled it by attached heterotrophs, showing the importance of attachment in transferring recently fixed carbon to secondary consumers. Hence, one can easily understand that any temperature changes going to affect the mutual as well as opportunistic bacterial associations with plants which will again also depend on the plant species. So, such kind of knowledge going to be the gold standard at the time of selecting the bacterial species to get the maximum benefit in the type of crop as well as high-temperature stress management.

### Plant-symbiotic bacteria interactions during salt stresses

A horrible situation encountered by the plants during the salt stress. Sessile plants experience many environmental stresses throughout their development. Salinity threatens 25%-33% of global crop production (Kumar et al. 2022). Salinity stress affects 85% of the planet, according to the FAO (Food and Agriculture Organization of the United Nation, 2022). Bad agricultural practices (high salt content water used for irrigation and fertilization) and saline water from the sea, rivers, etc., especially in arid and semiarid regions, cause soil salinity (Zhang et al. 2021). Climate change, drought, and rising sea levels often make soil saline (Kumar et al. 2022). High osmotic stress impairs plant nutrient uptake and transport (Farooq et al. 2015). Salinity stress negatively impacts plant physiological development (Mahawar and Shekhawat 2019) and soil microbiota also compromises the soil health (Dubey et al. 2022). yet, plants can capable of competing salinity stress by altering a variety of morphological, physiological, and molecular responses (Zhao et al. 2020), such as increasing phytohormone and osmoprotectant synthesis, antioxidant activity, Na+ homeostasis, and compartmentalization (Arif et al. 2020). Cohabiting with diverse halotolerant (HT) microorganisms improves plant growth, stress tolerance, and nutrient uptake, restoring crop yield (Etesami and Beattie 2018; Etesami and Glick 2020). Rhizosphere, epiphytes, and endophytes are the types of plant-interacting HT microorganisms (Andrews and Harris 2000; Hardoim et al. 2015). Accountable reports showed that plant species can colonize their self-bacterial populations (Kuske et al. 2002). Plants used three main strategies to survive in saline environments: osmotic Na^+^/Cl^-^ stress, exclusion, and accumulation. HT-PGPR, diverse saline soil microbes, improves plant adaptation to salinity stress. HT-PGPR increases the saline-agro-ecosystem productivity directly by producing exopolysaccharides (EPS), siderophores, volatile organic compounds (VOCs), compatible osmolytes, and phytohormones (Bhat et al. 2020) or indirectly by regulating stress-related gene expression and phytopathogen effects (Prasad et al. 2019b). *Rhizobium*, *Arthrobacter*, *Flavobacterium*, *Alcaligenes*, *Pseudomonas*, and *Azospirillumare* capable of improving the crop salinity tolerance (Saghafi et al. 2019; Kumar Arora et al. 2020), whereas, stressed HT bacteria form biofilms as well as produce EPSs (Haque et al. 2022).

The bacterial growth phase, nutrient medium composition, stress stimuli, pH, and temperature affect HT-PGPR EPS production (Kumar Arora et al. 2020). EPS makes up 40-90% of bacterial weight in saltwater (Sunita et al. 2020). The rhizosheath, which also acts as a carbon source, improves the nutrient and water uptake from the rhizosphere and protects the plant from ionic salts and phytopathogens (Mishra and Arora 2018; Kumar Arora et al. 2020). It was examined how EPS-producing halotolerant *Enteroba ctercloacae* and *B. drentensis* increased crop plant water uptake and nutrient availability to improve salt-stressed mung bean growth (Mahmood et al. 2016). EPS promotes soil aggregation, humification, water retention, nodulation, quorum sensing, and microbial diversity that protects plant cells from desiccation in saline environments (Kumar Arora et al. 2020). Antioxidants in EPS can protect against salinity-induced oxidative damage (Sunita et al. 2020). Volatile organic compounds (VOCs) are produced by HT-PGPR under stress (Sunita et al. 2020). VOCs stimulate siderophores, osmoprotectants, phytohormones, HKT1/K^+^ transporters, virulence factors, and bacterial motility in the plant-microbe interaction to help stressed plants grow and adapt (Bhat et al. 2020). Salinity-stressed crops also face low availability of soluble ferrous ions (Fe^2+^), which is mostly determined by soil pH. Alkaline pH (pH >6.5) in saline soils oxidises Fe^2+^ to ferric (Fe^3+^) and reduces plant iron availability. Salt-stressed crops' Fe needs are met by Fe^3+^ HT-PGPR's and siderophores. HT PGPR can help plants accumulate compatible osmolytes (amino acids, soluble sugars, and polyols) in a saline environment to reduce osmotic stress, and maintain high turgor pressure and cytoplasmic ion equilibrium. To reduce water stress in plants HT-PGPR upregulates the osmolyte biosynthesis genes (mainly proline), and controls stomatal conductance and transpiration. Bioinoculation of capsicum with a halo tolerant PGPR (B.fortis SSB21) increases the proline synthesis and stress-related gene expression including the pepper pathogen-induced protein gene (CAPIP2), putative ketoacyl-ACP reductase (CaKR1), pepper osmotic-like protein 1 (CaOSM1), and pepper class II basic chitinase (CAChi2). Salt-tolerant PGPR upregulates the phytohormone synthesis genes, mainly IAA, to compensate for plant growth hormones, change root morphology, and exclude excess ionic salts. In vitro studies showed that HT-PGPR increases IAA production in plants, which reduces tap root growth, elongates root hairs, and increases lateral root number and length. Thus, water and nutrient availability and uptake improve crop growth. In *Coleus forskohlii*, *P. putida* and *Maltophilia* increased IAA, gibberellic acid, and cytokinin production. Under NaCl stress, *Pseudomonas sp*. Increases the gibberellin and cytokinin production in *Glycine max* and *Zea mays* plants respectively. PGPR synthesizes and regulates stress hormones ABA and ethylene in addition to other growth hormones. HT strain *Dietzia natronolimnaea STR1*upregulates the expression of ABA signaling cascade genes like ABA response elements (TaABARE) and 12-oxophytodienoate reductase 1 (TaOPR1), which stimulates the salt stress-induced gene TaST in *T. aestivum* (Bharti et al. 2016). Ethylene, another stress hormone, increases salinity tolerance but limits plant growth and productivity. HT-PGPR secrete ACC deaminase, which metabolizes ACC (ethylene precursor) into alpha-ketoglutarate and ammonia, inhibiting plant ethylene synthesis (Bhat et al. 2020). Certainly, it has been indicated that the inoculation with plant growth promoting *Rhizobacteria* containing ACC-deaminase could be a useful approach for improving growth and yield of maize under salt-stressed conditions (Nadeem et al. 2009). In addition to producing plant-beneficial metabolites HT-PGPRs also regulate the Na^+^/K^+^ homeostasis and modulate the expression of salt-tolerant genes like salt overly sensitive (SOS), high-affinity K^+^ transporters (HKT), Na^+^/H^+^ antiporter (NHX), aquaporins (AQPs), and antioxidants to protect plants from salinity stress. HT-PGPR B. subtilis (GB03) reduces Na^+^ uptake in the halophyte grass *Puccinellia tenuiflora* by upregulating PtHKT1;5 and PtSOS1 and downregulate the PtHKT21 (Wang et al. 2015). In saline plant (*T. aestivum*) the bacteria *B. subtilis GB03* execute similar mechanism (Zhang et al. 2014). In salt-stressed *Zea mays*, HT-PGPR *B. megaterium* upregulates the aquaporin genes (ZmPIP1-1 and PIP2), increasing water uptake (Marulanda et al. 2010) by modulating antioxidant expression and activity, HT-PGPR increases host plant salt tolerance (Kumar Arora et al. 2020). The HT-PGPR boosts crop growth and yields in normal and stress conditions better than synthetic agrochemicals. Many PGPR-based bioformulations and products are in development or present in the market for applications. Laboratory screening assays and field trials are essential to PGPR production (Backer et al. 2018). Commercial PGPR inoculants did not promote crop growth in agricultural fields like those under controlled laboratory conditions. Climate change hinders field PGPR performance. Climate change affects plant physiology and plant-associated microbial community diversity, abundance, colonization, and activity independently as well as jointly (Tabassum et al. 2017). It is quite obvious that no potent commercial inoculant works in all ecological zones, therefore, climate also affects the PGPR efficiency (Liu et al. 2022). Bioinoculants must also consider crop variety and PGPR strain. PGPR strains or consortia affect crop growth and yield differently depending on the cultivar. PGPR performance depends on carrier choice. Inappropriate carriers reduce rhizosphere bacteria survival and efficiency. Another factor is microbe consortia compatibility. Many bacterial strains interact antagonistically, reducing field PGPR efficiency (Tabassum et al. 2017). Halophytes store plant-growth-promoting halotolerant bacteria. High phosphorus-solubilizing halotolerant PGPR bacteria (sp.YCWA18) isolated from the place Daqiao Kushneria saltern sediment on the eastern coast of China capable of growing on a solid medium with 20% (w/v) NaCl (Zhu et al. 2011). PGPRs that were found to be halotolerant based on their ability to tolerate 2-25% NaCl, including *B. pumilus*, *P. mendocin*, *Arthrobacter*, *Halomonas*, *Nitrinicola lacisaponensis*, and with other PGP traits like phosphorus (P) solubilization and the ability to produce IAA, siderophores, and ACC deaminase have also been isolated (Tiwari et al. 2011). Due to poor solubility and soil fixation, only 0.1% of P is available to plants (Goldstein 1986). Inorganic NPK fertilizers and saline irrigation increase soil salinity. Phosphate-solubilizing HT PGPRs increase plant P availability without increasing soil salinity. PGPRs can solubilize insoluble phosphates through chelation, ion exchange, and acidification by secreting low-molecular-weight organic acids (Sharma et al. 2013; Etesami 2018). Under field salinity stress, wheat inoculated with *B. aquimaris* plant increases P content (Upadhyay and Singh 2015).

### Plant-symbiotic bacteria interactions during drought stresses

Drought conditions in plants directly as well as indirectly affect the root bacterial communities by modulating moisture availability, soil chemistry, and plant phenotypes. Due to resource limitation, total bacterial biomass decreases under drought (Hueso et al. 2012; Alster et al. 2013) and in more arid soils along a precipitation gradient (Bachar et al. 2010). In moisture-limited soils, the presence of bacteria *Proteobacteria*, *Verrucomicrobia*, and *Bacteroidetes* decreased and the genera of *Firmicutes* and *Actinobacteria* found to be increased (Barnard et al. 2013; Bouskill et al. 2013; Acosta-Martínez et al. 2014; Curiel Yuste et al. 2014). The soil community composition shifts have several possible causes. First, Gram-positive and Gram-negative bacteria may have different drought susceptibilities due to substrate preference and metabolic capacities. Drought stress conditions can be oligotrophic-nutrient-poor but could be oxygen-rich. Oligotrophs (slow-growing microbes) usually grow in poor conditions. In droughted soils, oligotrophic bacteria proliferate because of complex plant polysaccharide degradation genes that are more abundant than those targeting oligosaccharides (Bouskill et al. 2016b; Martiny et al. 2017). Gram-positive bacteria are metabolically "hardy" and can use inorganic nitrogen to produce extracellular enzymes that degrade complex organic compounds, which are abundant in droughted soils (Treseder et al. 2011). *Actinobacteria* genera can use recalcitrant carbon sources and are abundant in arid, nutrient-poor soils (Connon et al. 2007; Curiel Yuste et al. 2014; Mohammadipanah and Wink 2016; Hartmann et al. 2017). Gram-negative bacteria are copiotrophs, preferring labile carbon compounds and organic nitrogen, especially in the form of plant root exudates (Balasooriya et al. 2014). Under well-watered conditions, Gram-negative bacteria incorporated almost ten times as much plant-derived carbon as Gram-positive bacteria (Fuchslueger et al. 2014). Plants mechanistically close the protein channels to prevent sugar transport to the rhizosphere during drought osmotic adjustment. Bacterial activity levels may be a third cause of drought-induced community composition changes. The "Birch effect" occurs after rewetting dry soils, where bacterial activity and CO_2_ efflux increase dramatically (Blazewicz et al. 2014; Armstrong et al. 2016). Wetter soils have less gaseous diffusion, creating a more anaerobic environment (Liptzin et al. 2011) and higher bacterial gene abundances for genes involved in anaerobic fermentation (Schwartz et al. 2007), O_2_ limitation, and denitrification (Bouskill et al. 2016b). Under drought, soil bacterial activity decreases as microbes die or go dormant (Jensen et al. 2003; Alster et al. 2013), but complex carbon degradation gene abundance increases (Bouskill et al. 2016b). In addition, drought can trigger presented microbes to fabricate a diversity of compounds that affect community stability. Drought encountering soils enriched in antibiotics, which drought-liberal bacteria may construct to suppress other bacteria for existing resources or as signals to enhance drought-encountering mechanisms like biofilm formation (Bouskill et al. 2016a). During drought, bacteria produce compounds that affect rhizosphere soil aggregate stability and hydrophobicity (Kohler et al. 2009). Finally, osmolytes maintain cellular turgor and protect macromolecular structures (Welsh 2000). Season has the biggest influence on the root endosphere microbiome of wild and cultivated *Agave* species, while host species influence the rhizosphere and leaf phyllosphere (Desgarennes et al. 2014; Coleman-Derr et al. 2016). *Cacti* and *Populusdeltoides* showed seasonal root endosphere influence (Shakya et al. 2013; Fonseca-García et al. 2016). In the wildflower genus Banksia (Marschner et al. 2005) and date palms, seasonal soil moisture changes affect the diversity of root and rhizosphere bacterial community (Cherif et al. 2015). The drought regime was the second-biggest beta-diversity factor in cereal grasses after the plant compartment (Naylor et al. 2017). The tree species *P. deltoids* (Shakya et al. 2013), *Mimosa tenuiflora* (Taketani et al. 2017), and the cactus *Cereus jamacaru* have rhizosphere communities that show increased abundance of *Actinobacteria*, *Acidobacteria*, and *Bacillus* in the dry season and *Proteobacteria* and *Bacteroidetes* in the rainy season (Nessner Kavamura et al. 2013). Generations of repeated drought events may have evolved stable and beneficial plant-microbe interactions that improve host and microbe reproductive fitness. In one study, *Brassica rapa* plants exposed to generations of drought were better than controls at increasing bacterial abundance and diversity around roots in dry contemporary environments Because microbes with PGP activities improve plant health and fitness, stable plant-microbiome interactions are an attractive target for crop stress tolerance (Quiza et al. 2015). Plants may select for certain microbial traits by studying drought-treated plant growth-promoting microbes. Rhizosphere isolates had more enzymes than the bulk soil isolates (Timmusk et al. 2011). In a pepper plant study, root endophytes dominated the phyto-hormone synthesis and rhizosphere whereas bulk soil dominated the nutrient solubilizers (Marasco et al. 2012). Only during drought conditions, PGPB can improve drought tolerance as well as plant growth. Bacterial consortia may reduce drought-induced stress better than the individual genera (Knoth et al. 2014; Timm et al. 2016). In a study, microbes have shown the initial positive effects on plant performance but negative effects during severe drought conditions (Redman et al. 2002). Plant physiological responses to severe drought can be greatly influenced by soil microbial communities during well-watered and moderate drought conditions (i.e., zero soil moisture). Soil microbes regulate soil moisture, nutrient availability, and plant function [1, 5]. *Azospirillum brasilense* increased ABA content in a microbe–ABA interaction study (Cohen et al. 2015). In *Oryza sativa*, endophytic bacteria increased SA and ABA concentrations, possibly improving stress tolerance (Shahzad et al. 2017). Interestingly, over-expression of certain stress genes has been observed in experimental conditions which elevate the expression of salicylic acid, and regulate plant growth and the immune responses (Devarajan et al. 2021). It was shown that *Pseudomonas putida* (FBKV2) inoculation increased the SnRK2 family proteins and ABA-responsive gene transcription (SkZ et al. 2018). A treasure of plant and symbiotic microbes is listed in Table 2.

**Table 2:** Role of symbiotic bacteria’s during encounter of abiotic stress in plants

**Abiotic stress**	**Mutualitstic** **bacteria**	**Host plant**	**Beneficial function**	**References**
**1. High Temperature**				
** (i)**	*Burkholderia phytofirmans strain PsJN*	*Tomato* *(Solanum lycopersicum)*	• Improve photosynthesis in PSJN strain of *P. phytofirmans* and promote growth at high temperature • Improve heat tolerance and prevent the starch degradation of starch in bacterized tomato plants also promote gas exchange, alleviate heat stress	(Issa et al. 2018)
** (ii)**	*Rhizobium species,* *Azotobacter*	*Soya bean*	• Fix atmospheric nitrogen by converting nitrogen into ammonia to make possible the availability of nitrogen to the crop	(Shokri and Emtiazi 2010)
** (iii)**	*Bacillus aryabhatti*	*Soybean*	• *Bacillus aryabhattai* bear oxidative and nitrosative stress and endorse the soyabean growth production and modulation of phytohormones	(Park et al. 2017)
**2. Salt**				
** (i)**	*Bacillus fortis SSB21*	*Capsicum*	• Improved proline synthesis and expression of stress related genes (pepper pathogen-induced protein gene (CAPIP2), putative ketoacyl-ACP reductase (CaKR1), pepper osmotin-like protein 1 (CaOSM1), and pepper class II basic chitinase (CAChi2) during salinity stress	(Yasin et al. 2018)
** (ii)**	*Enterobacter cloacae* *Bacillus drentensis*	*Mung bean*	• Increase the water uptake and nutrient availability in crop via EPS rhizosheath formation via EPS and improve the availability and nutrient from the rhizosphere	(Kumar Arora et al. 2020)
** (iii)**	*Bacillus fortis SSB21*	*Capsicum*	• Improve proline synthesis and expression of stress related genes (pepper pathogen-induced protein gene CAPIP2, putative ketoacy l-ACP reductase CaKR1, pepper osmotin-like protein 1 CaOSM1 and pepper class II basic chitinase CAChi2) during salinity stress	(Yasin et al. 2018)
** (iv)**	*Pseudomonas putida Pseudomonas stutzeri, Stenotrophomonas maltophilia*	*Coleus forskohlii*	• Enhance IAA, gibberellic acid, and cytokinin in plants	(Patel and Saraf 2017)
** (v)**	*Pseudomonas sp.*	*Glycine max* *Zea mays*	• Enhanced the production of gibberellins and cytokinin in plant	(Sandhya et al. 2010; Kang et al. 2014)
** (vi)**	*Dietzia natronolimnaea STR1*	*Triticum aestivum.*	• Induce the expression of ABA signaling cascade genes (ABA response elements TaABARE, and 12-oxophytodienoate reductase 1 TaOPR1) that stimulate the expression of the salt stress-induced gene (TaST. EE)	(Bharti et al. 2016)
** (vii)**	*Enterobacter spp., Pseudomonas fluorescens*	*Zea mays*	• Produce ACC and degrade ethylene	(Nadeem et al. 2009)
** (viii)**	*Bacillus subtilis (GB03)*	*Puccine lliatenuiflora*	• Reduces Na^+^ uptake via upregulation of PtHKT1;5 and PtSOS1 and downregulate the gene PtHKT2	(Niu et al. 2016)
** (ix)**	*Bacillus subtilis* *Arthrobacter species*	*Wheat*	• Abled salinity stress tolerance by adaptive evolution • Enhance the growth parameter • Increase production of biochemical’s • Reduce the effect of malondialdehyde and hydro.gen peroxide. • Modulate antioxidant enzyme activity. • Increasing the uptake of nutrient ability	(Gul et al. 2023)
** (x)**	*Serratiaplymuthica (KM 4)*	*Cucumis*	• Tolerance of salinity stress by regulation of redox potential, ion homeostasis, leaf gas exchange and stress-related gene expression	(El-Esawi et al. 2018)
** (x)i**	*Bacillus megaterium*	*Zea mays*	• Upregulate the expression of aquaporin genes (ZmPIP1-1 and PIP2) that increase the water uptake in salt-stressed plants	(Marulanda et al. 2010)
** (xii)**	*PGPRs*	*Haloxylon salicornicumfruticosa* *Lespedeza bicolor* *Atriplex leucoclada Salicorni cavirginica Suaeda*	• Enhance the growth of salinity stressed maize. • Increase in antioxidant enzymes activity (SOD, peroxidase, CAT and ascorbate peroxidase)	(Ullah and Bano 2015)
**3. Drought**				
** (i)**	*S. meliloti*	*M. sativ* *Alfalfa*	• Enable drought • Drought tolerance by synthesis of cytokines	(Xu et al. 2012)
** (ii)**	*Glucoacenatobacter diazotrophicus*	*Sugarcane*	• Activate the drought responsive gene	(Vargas et al. 2014)
** (iii)**	*Pantoea alhagi*	*Whea*	• Increase accumulation of soluble sugar • Decrease accumulation of proline and MDA • Decrease the degradation of chlorophyll in leaves of drought-stressed wheat	(Chen et al. 2017)
** (iv)**	*Pseudomonas aeruginosa*	*Vigna radiata*	• Increase production of IAA, ACC deaminase and siderophor	(Uzma et al. 2022)
** (v)**	*Varivorax paradoxus 5C-2*	*Pea,* *Maize*	• Regulates the abscisic acid signaling	(Dodd et al. 2009)
** (vi)**	*Bacillus subtilis* *Paenibacillus* *illinoinensis*	*Pepper*	• Drought toleranc	(Vigani et al. 2018)
** (viii)**	*Bacillus velezensis* *(B26)*	*Brachypo diumdistachyon*	• Upregulation of drought response genes DREB2B land modulation of DNA methylation genes	(Gagné-Bourque et al. 2015)

## Evident relations of viral pathogenesis and symbiotic bacteria in plants

Stress factors can compromise the plant's immune defenses rendering it susceptible to viruses. However, growth-promoting bacteria enhance plant growth by several factors. The above-mentioned root bacteria help plants against various biotic and abiotic stresses. These beneficial bacteria's are called "plant growth-promoting rhizobacteria" (PGPR). Viruses, on the contrary, were shown to have rare beneficial roles such as conferring resistance against various abiotic stresses. Abiotic stress like drought, salinity, and changing climatic temperatures trigger physiological responses that suppress plant's immune functions offering numerous advantages to opportunistic viruses. Contrarily, symbiotic bacteria alleviate the negative impact of abiotic stress and bolster the plant's immune capacity against infections. PGPRs have been shown to help plants against viral infections by inducing systemic resistance (Yu et al., 2022). It was shown that when plants are exposed to these bacteria, the immune system gets more active and fends off potential pathogens. Zehnder et al., 2000 reported that PGPRs induced systemic resistance (ISR) against *Cucumber mosaic cucumovirus* infection in tomatoes. Maurhofer et al., 1998 confirmed the expression of salicylic acid biosynthesis genes in *Pseudomonas fluorescens strain P3* that induced systemic resistance against *Tobacco necrosis virus* in tobacco plants. A jasmonic acid-dependent and SA-independent signalling pathway induced by PGPR isolates (*Serratia marcescens* strain 90-166 and *Bacillus pumilus* SE34) provides protection against CMV in *A. thaliana* (Ryu et al., 2004). Hence, PGPRs influence plant hormones level which in turn impact plant's immune responses against viral infections.

PGPRs can also produce certain compounds or enzymes that directly affect or inhibit the growth of viruses. A 39.4 KDa anticiotic protein has been identified and purified from the *P. fluorescens* strain CZ which has confirmed the 74.81% inhibitory effect against TMV (Shen et al, 2013). Some volatile organic compounds like 2,3-butanediol produced by bacteria *P. chlororaphis O6*, provided resistance against cucumber mosaic virus, tobacco mosaic virus, pepper mottle virus, and tomato yellow leaf curl virus in pepper (Kong et al., 2018). Application of *P. fluorescens* controlled *Squash mosaic virus* infection in cucumber (Firmansyah et al., 2017). Whereas Elbeshehy et al., 2015, showed resistance induction in pumpkins against watermelon mosaic virus by application of *B. subtilis* and *B. pumilus*. A large number of defense enzymes like Dicer-like (DCL) enzymes, binases, baRNases, peroxidase and phenylalanine ammonia-lyase etc. are activated by application of PGPRs on plants, inhibiting viruses through inactivation of their RNA or by strengthening the plant’s inborn immunity (Manjunatha et al., 2022). These bacteria mediate modulation of defense enzyme pathways that fine-tune the plant's response to both stress and viruses. The interplay between these factors contributes towards a harmonious defense mechanism that not only protects plants from abiotic stress but also makes plants resilient against viral infections. The research cites the importance and opportunities to harness the potential of root symbiotic bacteria for sustainable agricultural practices, ensuring robust crop protection in the face of changing environmental conditions.

## Conclusions and future directions

In conclusion, the present review has elaborated on the pathological outcomes of viral pathogens and beneficial outcomes of symbiotic bacteria in plants. The perspective on such interactions primarily revolves around examining viral pathogenesis under both (abiotic stress and natural environmental) conditions. Simultaneously, discussions have been presented regarding the beneficial role of symbiotic bacteria residing in the plant roots, which directly or indirectly contribute to plant growth promotion and help plants to cope-up with abiotic stress. However, limited literature is available to establish an evident relationship between "viral pathogenesis-abiotic stress-root microbes" conferring the respective harmful and beneficial outcomes, and this part was also discussed precisely. Hence the information gathered in the review is crucial to design future studies that shed light on the intricate association between pathogens, hosts, and symbionts, with a focus on plant/microbial biotechnology and understanding the dynamics of host-microbe relationships to achieve a balanced interplay between plants and bacterial microbes. Furthermore, there is a need for further exploration to better comprehend the role of mycorrhiza and their associated endophytes in enhancing plant immunity against pathogens, as well as their involvement in nutrient acquisition processes from the soil.

## Data Availability

Not applicable.

## Abbreviations

ACC oxidase: 1-Aminocyclopropane-1-carboxylic acid oxidase
BIK1: Botrytis-Induced Kinase1
DOC: Dissolved organic carbon
EPS: Exopolysaccharides
ETI: Effectors triggered immunity
HKT1: High affinity K transporter 1
HR: Hypersensitive response
HSPs: Heat shock proteins
HT: Halotolerant
MAPK: Mitogen activated protein kinases
NADP: Nicotinamide adenine dinucleotide phosphate
NBLRR protein: Nucleotide Binding Domain and Leucine-rich Repeat.
PCD: Plant cell death
PGPR: Plant growth-promoting rhizobacteria
PR genes: Pathogenesis Related genes
PTI: Pattern triggered immunity
R-gene: Resistance genes
ROS: Reactive oxygen species
siRNAs: Small interfering RNAs
SMs: Secondary Metabolites
SOS: Salt overly sensitive
VOCs: Volatile organic compounds
vsiRNAs: Viroid derived small interfering RNAs
